# A Flexible and Wearable Nylon Fiber Sensor Modified by Reduced Graphene Oxide and ZnO Quantum Dots for Wide-Range NO_2_ Gas Detection at Room Temperature

**DOI:** 10.3390/ma15113772

**Published:** 2022-05-25

**Authors:** Qijing Lin, Fuzheng Zhang, Na Zhao, Libo Zhao, Zuowei Wang, Ping Yang, Dejiang Lu, Tao Dong, Zhuangde Jiang

**Affiliations:** 1 State Key Laboratory for Manufacturing Systems Engineering, School of Mechanical Engineering, Xi’an Jiaotong University, Xi’an 710049, China; xjjingmi@163.com (Q.L.); zn2015@stu.xjtu.edu.cn (N.Z.); libozhao@xjtu.edu.cn (L.Z.); wzw437@stu.xjtu.edu.cn (Z.W.); ipe@xjtu.edu.cn (P.Y.); djlu@xjtu.edu.cn (D.L.); zdjiang@mail.xjtu.edu.cn (Z.J.); 2 Chongqing Key Laboratory of Micro-Nano Systems and Intelligent Sensing, Chongqing Academician Workstation, Chongqing 2011 Collaborative Innovation Center of Micro/Nano Sensing and Intelligent Ecological Internet of Things, Chongqing Technology and Business University, Chongqing 400067, China; tao.dong@usn.no; 3 School of Mechanical and Manufacturing Engineering, Xiamen Institute of Technology, Xiamen 361021, China

**Keywords:** reduced graphene oxide, ZnO quantum dots, NO_2_ gas sensor, flexible and wearable, early warning system

## Abstract

Reduced graphene oxide (rGO) fiber as a carbon-based fiber sensor has aroused widespread interest in the field of gas sensing. However, the low response value and poor flexibility of the rGO fiber sensor severely limit its application in the field of flexible wearable electronics. In this paper, a flexible and wearable nylon fiber sensor modified by rGO and ZnO quantum dots (QDs) is proposed for wide-range NO_2_ gas detection at room temperature. The response value of the nylon fiber sensor to 100 ppm NO_2_ gas is as high as 0.4958, and the response time and recovery time are 216.2 s and 667.9 s, respectively. The relationship between the sensor’s response value and the NO_2_ concentration value is linear in the range of 20–100 ppm, and the fitting coefficient is 0.998. In addition, the test results show that the sensor also has good repeatability, flexibility, and selectivity. Moreover, an early warning module was also designed and is proposed in this paper to realize the over-limit monitoring of NO_2_ gas, and the flexible sensor was embedded in a mask, demonstrating its great application potential and value in the field of wearable electronics.

## 1. Introduction

With the development of modern industry, the types of toxic and harmful gases produced in production activities are becoming more and more diverse, especially the nitrogen dioxide (NO_2_) gas produced in the chemical production process, which poses a great threat to people’s lives and property [1]. Therefore, it is necessary to develop high-performance gas sensors for NO_2_ gas detection. NO_2_ gas sensors that can work at room temperature are particularly important because they have the advantages of low power consumption, no heating, and easy operation [2,3]. So far, many different types of NO_2_ gas sensors that work at room temperature have been manufactured by using different sensitive materials [4,5,6,7,8]. Among them, wearable NO_2_ gas sensors have the characteristics of flexibility, a light weight, and a low cost and important application potential and value in the field of wearable electronics [9,10,11].

rGO is a potential gas-sensing material. On the one hand, rGO’s conductivity at room temperature allows it to work at room temperature, and, on the other hand, its large surface area provides many active sites for gas sensing [12,13,14,15,16]. Reddeppa et al. [17] spin-coated an rGO layer on gallium nitride nanomaterials (GaN NRs) for improving the H_2_ and H_2_S gas sensing properties. The experimental results suggested that rGO is a promising material for improving the performance of GaN-based gas sensors. Metal oxide semiconductor materials, such as ZnO [18,19], SnO_2_ [20,21], CuO [22,23], and Ag_2_O_3_ [24,25,26], have a broad range of application prospects in the field of flexible gas sensors with the advantages of a simple preparation method, a controllable morphology, environmental friendliness, a low price, and stable physical and chemical properties. Mokrushin et al. [27] synthesized highly dispersed ZnO powders doped with europium and praseodymium by the solvothermal method. The morphology of the obtained nanopowders is nanorods with an average length of 67–82 nm, and the resulting ZnO-based films showed a high and selective response to 4–100 ppm NO_2_ at temperatures of 125–150 °C. Combining rGO with a metal oxide is also an important strategy for improving the room-temperature gas sensing performance. For example, Liu et al. [28] proposed a NO_2_ gas sensor based on a three-dimensional graphene aerogel and SnO_2_ nanomaterials. The response value of the sensor to 50 ppm NO_2_ gas is 0.065, and the response time and recovery time are 190 s and 224 s, respectively. The sensor’s response value to 200 ppm NO_2_ is also only 0.12. In addition, they also designed a NO_2_ gas sensor mixed with ZnO nanoparticles based on a graphene aerogel. The test results showed that the sensor has a faster response speed and a shorter recovery time, but the response value is only 0.089 for 100 ppm NO_2_ [29]. Based on graphitic carbon nitride, silver nanoparticles, and rGO, Qui et al. [30] designed and manufactured a sensor for detecting toxic gases. The sensor has a high response value to NO_2_ gas at room temperature, but the recovery is very poor, which is not conducive to practical application.

Materials whose three-dimensional scales are less than 100 nm are called nanoparticles, and when the particle radius is less than its exciton Bohr radius, the particles can be called quantum dots (QDs). It can be said that QDs are small-sized nanoparticles [31,32]. QDs have become a research hotspot in the field of gas sensors due to their multi-exciton effect, small size effect, surface effect, and tunnel effect. In particular, their small size enables them to have a larger specific surface area, thereby providing more active adsorption sites for gases [33]. Using a simple synthetic strategy, Liu et al. [34] fabricated a NO_2_ gas sensor based on MoS_2_ nanosheets decorated with PbS QDs. Compared with pristine MoS_2_ nanosheets, the response time and recovery time of the sensor are significantly improved. Purbia et al. [35] prepared a zero-dimensional nitrogen-doped graphene/SnO_2_ QDs heterostructure by a simple wet chemical method, and the test results showed that the sensor has a high response to ultra-low concentrations of NO_2_.

Therefore, combining rGO with QDs is an effective means by which to develop flexible nitrogen dioxide gas sensors with a high response value and good recoverability. In this work, a flexible sensor with rGO and ZnO QDs was designed and fabricated for detecting a wide range of NO_2_ gas concentrations. Nylon fiber was used as the sensor substrate for better flexibility. Test experiments were carried out to explore the sensor’s performance. The results show that the sensor can detect a wide range of NO_2_ gas concentrations with high response and gas selectivity values, short response and recovery times, and good flexibility. The sensor’s sensitivity mechanism to NO_2_ gas is also discussed and explained. In addition, a gas over-limit warning device was also designed and manufactured. Finally, the warning module was combined with the nylon fiber sensor embedded in a mask to carry out research on the wearable application to NO_2_ gas.

## 2. Materials and Methods

### 2.1. Materials and Gas Sensor Fabrication

**Figure 1:**
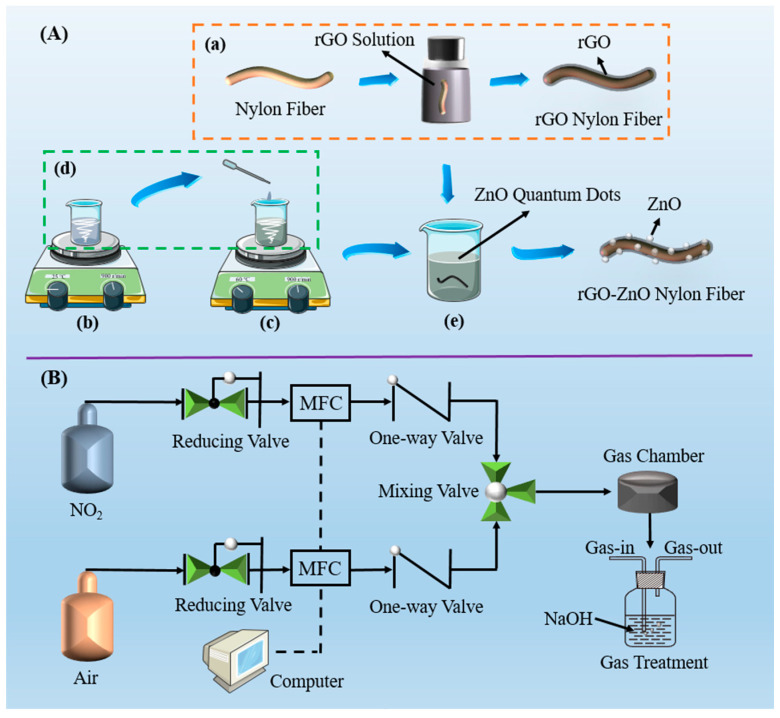
(**A**) Preparation process of the rGO/ZnO nylon fiber sensor: (**a**) preparation process of the rGO nylon fiber sensor; (**b**) the KOH solution; (**c**) the Zn(CH_3_COO)_2_·2H_2_O solution; (**d**) dropping the KOH solution into the Zn(CH_3_COO)_2_·2H_2_O solution; (**e**) the ZnO QDs solution. (**B**) Schematic diagram of the sensor testing device.

The preparation process of the rGO/ZnO nylon fiber sensor is shown in Figure 1A. The rGO was prepared by thermal reduction [36]. The rGO powder was added to ultrapure water to prepare a 0.5 wt% rGO solution, and then it was sonicated for 2 h through an ultrasonic machine to uniformly disperse the rGO. The nylon fiber with a diameter of 1 mm was put into ultrapure water for ultrasonic treatment to remove surface impurities such as dust. Then, the dried nylon fiber was put into a 0.5 wt% rGO solution and left to stand for 72 h. Ultrasonic treatment was also carried out in the intermediate process. The rGO nylon fiber was dried at room temperature (25 °C). Finally, a certain length of rGO nylon fiber was cut, with copper wires being wound around both ends of it, to obtain the rGO nylon fiber sensor. Different concentrations of ZnO QDs solutions (3 mM/L, 5 mM/L, 7 mM/L, and 9 mM/L) were prepared through zinc acetate dehydrate (Zn(CH_3_COO)_2_·2H_2_O), potassium hydroxide (KOH), and anhydrous methanol. As an example, the preparation process of the 5 mM/L ZnO QDs solution was as follows: (a) 0.0439 g Zn(CH_3_COO)_2_·2H_2_O was dissolved in 20 mL of anhydrous methanol and magnetically stirred at 70 °C for 20 min (solution A); (b) 0.0336 g of KOH was dissolved in 20 mL of anhydrous methanol and magnetically stirred at room temperature for 20 min (solution B); and (c) solution A and solution B were mixed and magnetically stirred for 2 h in order to produce the full reaction to obtain the 5 Mm/L ZnO QDs solution. Four rGO nylon fibers were put into the 3 Mm/L, 5 Mm/L, 7 Mm/L, and 9 Mm/L ZnO QDs solutions for 2 min to form rGO/ZnO-3, rGO/ZnO-5, rGO/ZnO-7, and rGO/ZnO-9 nylon fiber, respectively. The rGO/ZnO nylon fibers were also dried at room temperature. The method for manufacturing the rGO/ZnO nylon fiber sensors was the same as that for manufacturing the rGO nylon fiber sensor.

### 2.2. Characterization

Scanning electron microscopy (SEM, SU-8010, Tokyo, Japan) was used to observe the surface morphology of the nylon fiber sensor. The crystal structure of the material was determined by X-ray diffraction (XRD, D8 ADVANCE A25, Berlin, Germany) to analyze the diffraction pattern. An X-ray photoelectron spectrometer (XPS, Thermo Fisher ESCALAB Xi+, Waltham, MA, USA) was used to analyze the element composition in the sensor’s sensitive material. The Raman spectrometer (HORIBA, Shanghai, China) mainly used the Raman shift to determine the molecular structure of the substance and can realize qualitative analysis of samples. The semiconductor device analyzer (B1500A, Keysight, Petaling Jaya, Malaysia) was a test instrument that integrated a variety of measurement and analysis functions, and it was used to measure the I–V characteristics of the nylon fiber sensor.

### 2.3. Gas Sensor Test and Evaluation Index

The gas testing system is shown in Figure 1B. Two mass flow controllers (CS200, Sevenstar, Xi’an, China) were used to modulate the target gas with dry air as the carrier gas. After the target gas passed through the test chamber, it entered a gas bottle containing NaOH solution to complete the exhaust gas treatment. The resistance signal of the flexible sensor was collected by a data acquisition system (Keysight 34461A), and the obtained data were stored on a computer through the corresponding software. The response value (*S*) of the sensor was defined as (|*R*_0_ − *R*_a_|/*R*_0_) *×* 100%, where *R_a_* and *R*_0_ are the sensor’s resistance in the target gas and air, respectively. After the sensor comes into contact with the target gas, the time required for the resistance to reach 90% of the maximum resistance (*R_g_*) is defined as the response time (*T_res_*). The recovery time (*T_rec_*) was defined as the time required for the sensor’s resistance to recover from *R_g_* to *R*_0_ + 0.1 × |*R_g_* − *R*_0_| after the target gas is removed. Repeatability refers to the ability of a gas sensor to reproduce results within a certain period of time. Selectivity is the ability of a gas sensor to selectively respond to a specific target gas. It is generally measured by the response value of different gases at the same concentration. Linearity (*R*^2^) refers to the degree of linearity between the gas concentration and the sensor’s response value. Generally, the higher the linearity is, the better the sensor’s performance will be. The test environment’s temperature was around 25 °C.

## 3. Results and Discussion

### 3.1. Characterization of the rGO/ZnO Nylon Fiber Sensor

**Figure 2:**
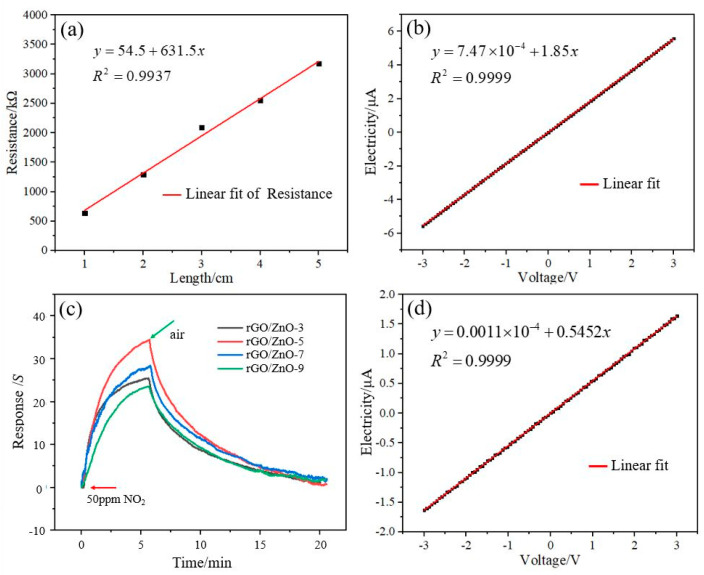
(**a**) Length and resistance relationship curve of the rGO nylon fiber; (**b**) I–V characteristic curve of the rGO nylon fiber sensor; (**c**) NO_2_ gas sensing performance curve of nylon fiber sensors with different ZnO QDs contents; and (**d**) I–V characteristic curve of the rGO/ZnO-5 nylon fiber sensor.

The relationship between the length and resistance of the rGO nylon fiber is linear, as shown in Figure 2a. The resistance of the rGO nylon fiber increases as the length increases. Its length ranges from 1 cm to 5 cm, and its resistance ranges from 641 kΩ to 3172 kΩ. The linearity is 0.9937, which shows that rGO is evenly distributed on the surface of the nylon fiber. The I–V characteristic of the rGO nylon fiber sensor is shown in Figure 2b. The resistance of the rGO nylon fiber sensor is 540 kΩ, and the test results show that it has good conductivity. rGO is a near-zero bandgap semiconductor with good electrical conductivity. The higher the degree of reduction, the closer it is to the properties of a metal, and the better the conductivity [37,38]. Therefore, the I–V test results show that the linearity of I and V is as high as 0.9999, which also indicates that the degree of reduction of the rGO is higher. The rGO/ZnO nylon fiber sensors with different ZnO QDs contents were tested for 50 ppm NO_2_ gas, as shown in Figure 2c. The response values of the four sensors are 25.4%, 34.4%, 28.2%, and 23.6%, respectively. The response value of the rGO/ZnO nylon fiber sensor is related to the number of ZnO QDs attached to its surface. The sensor’s response value first increases and then decreases with the increase in the number of ZnO QDs. A proper number of ZnO QDs can help improve the adsorption capacity of the sensitive film to NO_2_ gas, but an excessive number of ZnO QDs will reduce its adsorption capacity instead. The rGO/ZnO-5 nylon fiber sensor has the best sensing characteristics for NO_2_ gas. The I–V characteristic curve of the rGO/ZnO-5 nylon fiber sensor is shown in Figure 2d. The resistance of the rGO/ZnO-5 nylon fiber sensor is 1834 kΩ, which is greater than the resistance value (540 kΩ) of the rGO nylon fiber sensor. Because ZnO is a typical semiconductor, the conductivity effect is far less than that of rGO, and the surface of the rGO nylon fiber to which the ZnO is attached will increase the resistance of the sensor.

**Figure 3:**
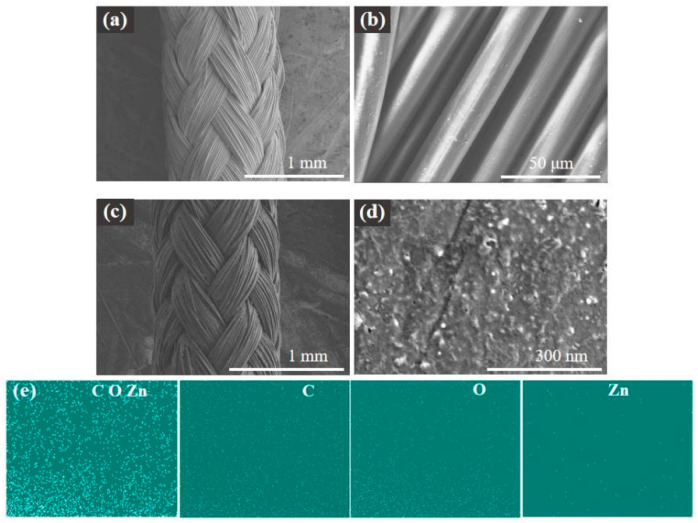
SEM images of (**a**,**b**) the nylon fiber and (**c**,**d**) the rGO/ZnO-5 nylon fiber sensor. (**e**) An EDS image.

**Figure 4:**
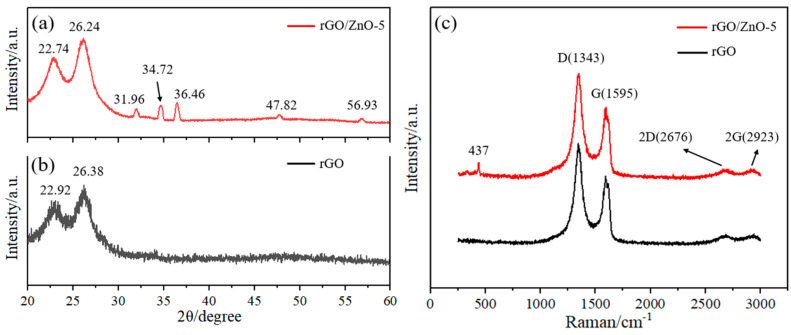
XRD spectra of (**a**) the rGO/ZnO-5 nylon fiber and (**b**) the rGO nylon fiber. (**c**) Raman spectra of rGO and rGO/ZnO-5 nylon fibers.

The surface morphologies of the nylon fiber are shown in Figure 3a,b. It is a white rope made of nylon material with flexible characteristics. As shown in Figure 3c,d, compared with the white nylon fiber, the color of the rGO/ZnO-5 nylon fiber is black because of the rGO. The ZnO QDs are also white and have a particle size of about 10 nm. They are evenly distributed on the surface of the rGO/ZnO-5 nylon fiber sensor. The EDS image of the rGO/ZnO-5 sensitive film is shown in Figure 3e. C, O, and Zn elements can be clearly observed, which further indicates that the ZnO quantum dots had been successfully prepared. Among them, the distribution of Zn is relatively sparse and uniform, indicating that the ZnO quantum dots are relatively uniformly dispersed on the surface of the rGO nylon fibers. XRD spectra of the rGO/ZnO-5 nylon fiber and the rGO nylon fiber are shown in Figure 4a,b, respectively. The diffraction peaks at 22.92°, 26.38°, 22.74°, and 26.24° are all caused by the nylon fiber substrate. The rGO/ZnO-5 sample has obvious diffraction peaks at positions 31.96°, 34.72°, 36.46°, 47.82°, and 56.93°, corresponding to the (100), (002), (101), (102), and (110) crystal planes of the wurtzite ZnO crystal structure, respectively (JCPDS File No.36-1451) [39], which indicates that ZnO QDs had successfully attached to the sensor surface. Raman spectra of rGO and rGO/ZnO-5 nylon fibers are shown in Figure 4c. The D and G peaks of rGO appear around 1343 cm^−1^ and 1595 cm^−1^, respectively. D is a defect peak, which represents the defective and disordered structure of the material, while G is generated by the vibration of carbon atoms hybridized by the Sp^2^ orbital [40]. The weak peak at 437 cm^−1^ is caused by ZnO [41]. The characteristic peaks of rGO and ZnO can be seen in the Raman spectrum of the sample, which provides further evidence of the formation of rGO/ZnO nanocomposites.

**Figure 5:**
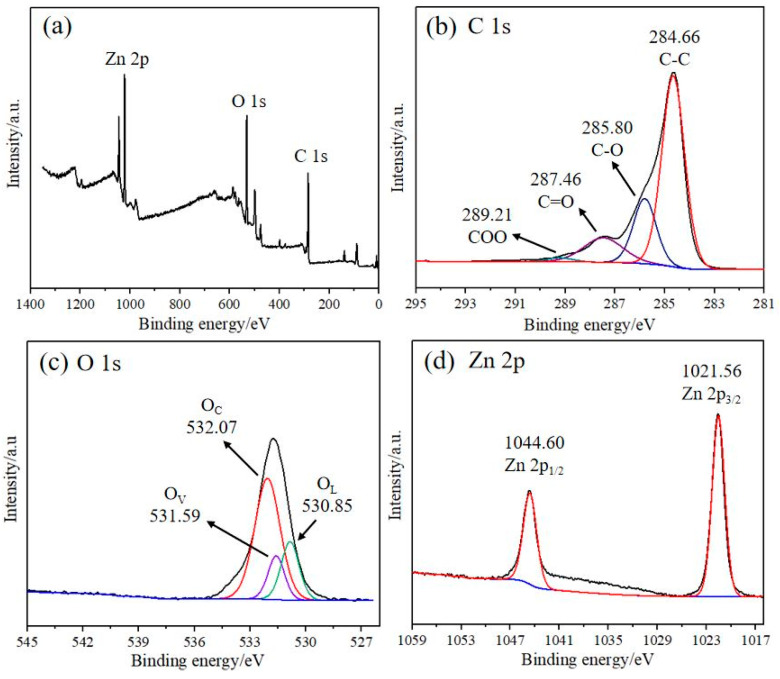
XPS spectra of (**a**) the rGO/ZnO-5 nylon fiber, (**b**) C 1s, (**c**) O 1s, and (**d**) Zn 2p.

XPS technology was used to qualitatively and quantitatively analyze the chemical elements of the rGO/ZnO-5 nylon fiber sample. The full spectrum of the rGO/ZnO-5 sample is shown in Figure 5a. The C, O, and Zn peaks can clearly be observed without other characteristic peaks, indicating that there are no other impurities on the fiber surface. Figure 5b shows the C 1s spectrum of the sample. Four peaks appear at positions 289.21 eV, 287.46 eV, 285.80 eV, and 284.66 eV, corresponding to COO, C=O, C-O, and C-C [42]. The peak of C-C is significantly higher than the other peaks, indicating that rGO had been successfully prepared. The O 1s spectrum is shown in Figure 5c. The three characteristic peaks of 531.59 eV, 532.07 eV, and 530.8 eV respectively correspond to the area of oxygen vacancies (O_V_), the surface-adsorbed oxygen components (O_C_), and the crystal oxygen substances (O_L_) [43,44,45]. The characteristic peak area of O_C_ is much higher than those of O_V_ and O_L_, indicating that the sensitive material has a strong ability to absorb and ionize oxygen, which is conducive to improving the gas sensing performance. Figure 5d shows the two characteristic peaks of Zn 2p (1044.60 eV and 1021.56 eV), which correspond to Zn 2p_1/2_ and Zn 2p_3/2_, respectively [46]. Taken together, these results prove that the composite materials that formed on the surface of the nylon fiber are rGO and ZnO, and further confirm the conclusions drawn from the XRD and Raman spectra.

### 3.2. NO_2_ Gas Sensing Test and Analysis of the rGO/ZnO-5 Nylon Fiber Sensor

**Figure 6:**
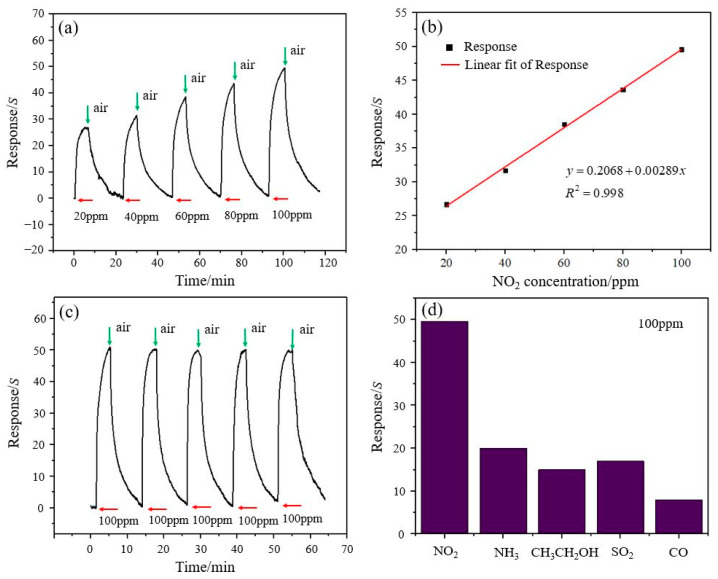
(**a**) The response value change curve of the rGO/ZnO-5 nylon fiber sensor in the NO_2_ concentration range of 20–100 ppm, (**b**) the linear fitting curve of the response values, (**c**) the repeatability test curve for 100 ppm NO_2_, and (**d**) the selectivity test results for different gases with a concentration of 100 ppm.

The test curve of the rGO/ZnO-5 nylon fiber sensor to NO_2_ gas at room temperature is shown in Figure 6a. From 20 ppm to 100 ppm NO_2_, the sensor’s response time and recovery time are similar. The response value of the sensor is able to recover to within 10% of the total response value when the NO_2_ concentration is as high as 100 ppm. For 100 ppm NO_2_ gas, the response time and recovery time are 216 s and 668 s, respectively. As shown in Figure 6b, the sensor’s response values increase linearly with the increase in the NO_2_ gas concentration and are 26.7% (20 ppm), 31.7% (40 ppm), 38.5% (60 ppm), 43.6% (80 ppm), and 49.6% (100 ppm). The linearity is as high as 0.998, indicating that the sensor has high practical value. Figure 6c shows the five-cycle test results of the sensor for 100 ppm NO_2_. The response value does not change significantly, which shows that the sensor has good repeatability. In addition, a gas selectivity test of the sensor was also performed, as shown in Figure 6d. The concentration of all the different gases was 100 ppm. Compared with ammonia (NH_3_), ethanol vapor (CH_3_CH_2_OH), sulfur dioxide (SO_2_), and carbon monoxide (CO), the response value of the sensor to NO_2_ gas is significantly higher than that of the other gases, indicating that the sensor has high selectivity to NO_2_ gas. In addition, humidity can affect the performance of flexible gas sensors. For NO_2_ gas sensors based on an rGO/metal oxide composite, in general, the sensor’s response will decrease as the humidity increases [47,48].

**Figure 7:**
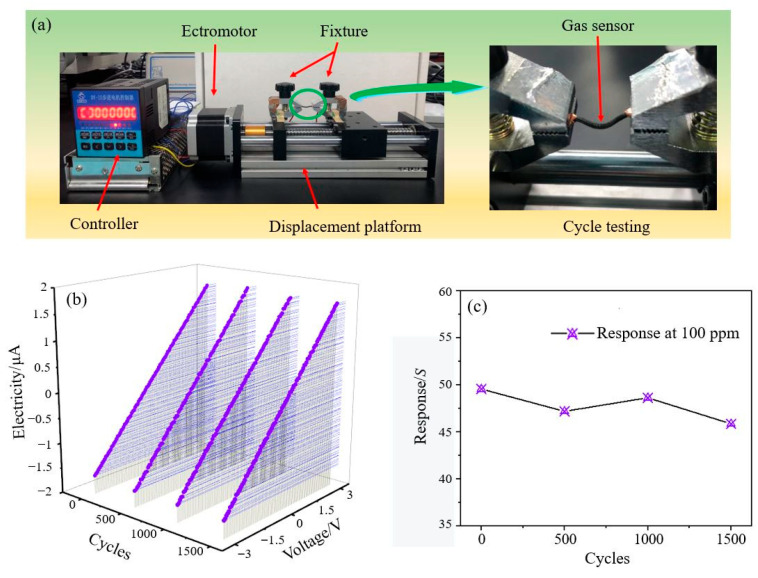
(**a**) The flexibility test device of the sensor, (**b**) the I–V characteristic curve, and (**c**) the response curve of the sensor with different numbers of bending cycles.

A flexibility test of the rGO/ZnO-5 nylon fiber sensor was also performed. Figure 7a shows the flexibility test device. The sensor’s I–V characteristic test results after 500, 1000, and 1500 bending cycles are shown in Figure 7b. The good linear relationship shows that the conductivity of the sensor is still very good after many bending cycles, and the resistance remained basically unchanged. For 100 ppm NO_2_, the response values of the sensor after 500, 1000, and 1500 bending cycles are 47.2%, 48.7%, and 45.9%, respectively. As shown in Figure 7c, compared with the response value (49.6%) without bending, they exhibited no significant changes, which shows that the sensor has good flexibility.

Table 1 shows a comparison between the sensing performance of our NO_2_ sensor and that of other reported gas sensors. Generally speaking, metal oxide gas sensors have short response and recovery times to gas at high temperatures [49], but the high-temperature heating condition also has obvious disadvantages, such as high power consumption, a lack of flexibility, and a limited application range. Guo et al. used laser micro-nanofabrication technology to fabricate a graphene-based NO_2_ sensor with short response and recovery times, but the sensor exhibited an obvious baseline drift phenomenon. Moreover, the sensor substrate is a rigid ceramic structure, which is not flexible and cannot be applied in the wearable field [50]. Moreover, compared with other flexible gas sensors that work at room temperature [51,52,53], the gas sensor has great advantages in terms of response value, response time, and recovery time. In particular, on the premise of better response and recovery times, the sensor has a high response value and no baseline drift, which are of great value to the practical application of the sensor.

**Figure 8:**
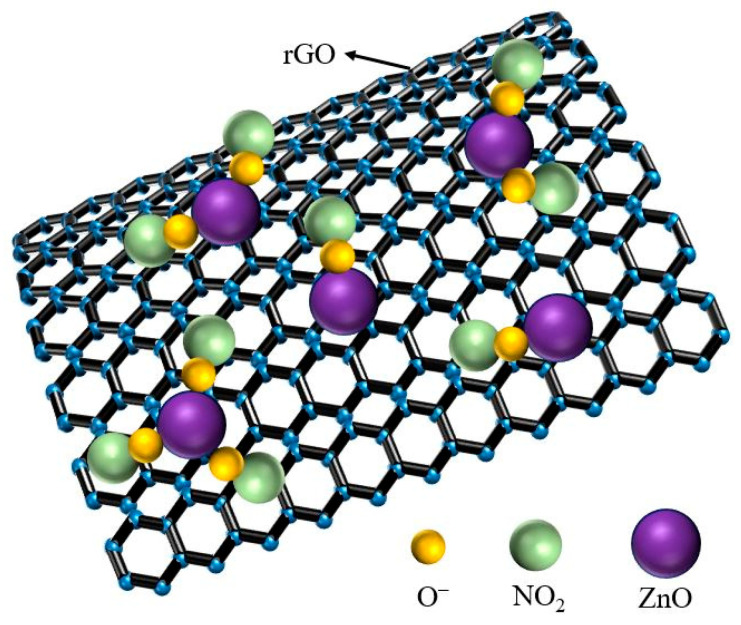
Schematic diagram of the gas sensing mechanism.

The gas sensing mechanism of the rGO/ZnO-5 nylon fiber sensor should follow the surface charge model shown in Figure 8. The rGO has a high electron migration rate at room temperature, and the rGO prepared in the experiment has a multilayer structure [53], which further promotes the transmission of electrons. On the surface of the rGO/ZnO nylon fiber, free electrons in ZnO will flow to the rGO, causing the composite’s resistance to increase [54]. This is the reason why the resistance of the rGO/ZnO-5 nylon fiber sensor is higher than that of the rGO nylon fiber sensor. When the sensor is exposed to NO_2_ gas with strong oxidizing characteristics, the free electrons in the ZnO will be taken away by NO_2_ gas molecules to create a ZnO electron depletion layer, which causes electrons to return from the rGO to the ZnO. The concentration of holes in the rGO will increase, thereby increasing the electrical conductivity of the sensor. In addition, NO_2_ gas molecules will also directly take away free electrons from the rGO, which further increases the concentration of holes in the rGO [55]. This will also improve the electrical conductivity of the sensor. Moreover, ZnO QDs are uniformly and densely distributed on the rGO layer, providing more reactive sites for the adsorption of NO_2_ gas molecules. This will further accelerate the rate of electron transfer, thereby improving the sensor’s response to NO_2_ gas.

### 3.3. Over-Limit Warning Module and Wearable Application

**Figure 9:**
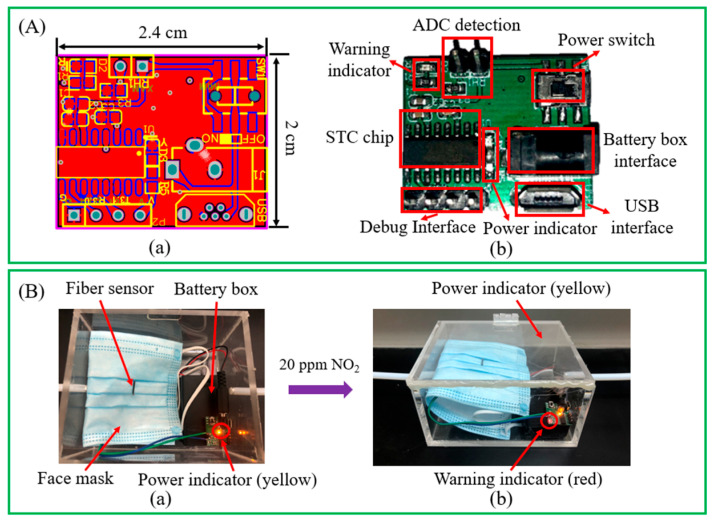
(**A**) Over-limit warning node module: (**a**) PCB; and (**b**) image of the physical system. (**B**) Working condition of the wearable sensor’s early warning system in (**a**) a normal gas environment; and (**b**) 20 ppm NO_2_ gas.

The over-limit warning module is shown in Figure 9A. The overall size of the node module is 2.4 cm × 2 cm. The small size facilitates its application in the field of wearable electronics. The warning module has seven main parts, namely an ADC detection module, a power switch, a warning indicator, an STC chip, a debug interface module, a power indicator, and a power supply module. The power module includes two power supply forms: battery and USB, which can be flexibly selected according to actual application scenarios. The power indicator is yellow, and the warning indicator is red. The nylon fiber sensor can be embedded in a mask because of its flexibility, which greatly promotes its application in the field of wearable electronics. Figure 9B shows the test experiment on the wearable sensor’s over-limit warning system. The alarm concentration of the over-limit warning module for NO_2_ gas was set to 20 ppm. In a normal environment, the power indicator is yellow but the warning indicator is not bright, indicating that the sensor’s power supply and NO_2_ concentration are normal. When 20 ppm NO_2_ gas is input into the environment, the warning indicator turns red, which shows that the sensor system can perform the warning function.

## 4. Conclusions

In summary, a flexible NO_2_ gas sensor (rGO/ZnO-5 nylon fiber sensor) that can work at room temperature was designed and fabricated with the advantages of a high response value, a high response speed, and good recoverability. Nylon fiber was used as the substrate, which greatly improved the flexibility of the sensor. rGO and ZnO QDs were used as sensitive materials to obtain high selectivity and good repeatability to NO_2_ gas. The gas sensing mechanism of rGO and ZnO QDs composites was also revealed. There is a highly linear relationship between the rGO/ZnO-5 nylon fiber sensor’s response and the NO_2_ gas concentration in the range of 20–100 ppm. In addition, an over-limit warning module was also designed and fabricated. The nylon fiber sensor was embedded in a mask, and its application in the field of wearable electronics was preliminarily tested.

## Figures and Tables

**Table 1:** Comparison between the sensing performance of our NO_2_ sensor and that of other reported gas sensors.

Sensor Structure	NO_2_ (ppm)	Temp (°C)	Response (S)	T_res_/T_rec_ (s)	Baseline Drift	Ref.
Zn_2_SnO_4_ film	40	200	29.3 R_g_/R_0_	8/58	No	Patil et al. [49]
RGO (two-beam laser interference)	20	RT	27% △R/R_0_	10/7	Yes	Guo et al. [51]
ZnO/graphene aerogel	100	RT	8.9% △R/R_0_	~500/~500	Yes	Liu et al. [29]
rGO/ZnO flowers and nanoparticles	1.5	RT	1.4% △R/R_0_	405/760	No	Adu et al. [51]
rGO/ZnO nanorods	100	RT	17.4% (△R/R_0_)	780/1980	Yes	Jing et al. [52]
rGO/ZnO QDs	100	RT	49.6% △R/R_0_	216/668	No	This work

## Data Availability

Data sharing is not applicable to this article.
